# The Multiple Sclerosis Intimacy and Sexuality Questionnaire (MSISQ-15): translation, adaptation and validation of the Polish version for patients with multiple sclerosis and spinal cord injury

**DOI:** 10.1186/s12883-021-02132-9

**Published:** 2021-03-08

**Authors:** Mikolaj Przydacz, Tomasz Golabek, Przemyslaw Dudek, Piotr Chlosta

**Affiliations:** Department of Urology, Jagiellonian University Medical College, ul. Jakubowskiego 2, 30-688 Krakow, Poland

**Keywords:** Multiple sclerosis, Spinal cord injury, Sexual dysfunction, Polish language, Questionnaire

## Abstract

**Background:**

Polish physicians and researchers lack an extensive and precise instrument in their native language for evaluating sexual dysfunction in individuals with neurogenic disorders. The aim of this study was to create a culturally adapted, validated, Polish language version of the Multiple Sclerosis Intimacy and Sexuality Questionnaire (MSISQ-15) for persons with multiple sclerosis (MS) and spinal cord injury (SCI).

**Methods:**

International recommendations and standardized methods for instrument validation were followed. Sexually active patients with MS and SCI completed the MSISQ-15, International Index of Erection Function (IIEF-15, men), and Pelvic Organ Prolapse/Urinary Incontinence Sexual Questionnaire (PISQ-31, women). IIEF-15 and PISQ-31 were used as reference questionnaires. Responses were collected at baseline (test) and after 2 weeks (re-test).

**Results:**

We recruited 299 Polish-speaking patients with MS or SCI. Interviews disclosed that the translated questionnaire had optimal content validity/cross-cultural adaptation. MSISQ-15 scores correlated significantly with the severity of sexual dysfunction as evaluated by IIEF-15 (*r* = − 0.487) and PISQ-31 (*r* = − 0.709). These correlations substantiated the high quality construct/criterion validity. An analysis of reliability presented good internal consistency (Cronbach’s alpha of 0.93 for the total score of MS patients and 0.86 for the total score of SCI patients) and reproducibility (intraclass correlation coefficients of 0.91 for the total score of MS patients and 0.92 for the total score of SCI patients). There were no ceiling or floor effects.

**Conclusions:**

The Polish version of MSISQ-15 exhibited excellent measurement properties. It is a suitable and reliable instrument to assess sexual dysfunction in MS and SCI individuals. The Polish MSISQ-15 will enhance routine clinical practice and assist research for neurogenic patients in Poland.

**Supplementary Information:**

The online version contains supplementary material available at 10.1186/s12883-021-02132-9.

## Background

Sexual functioning is a part of life’s vitality. Chronic neurogenic disorders such as multiple sclerosis (MS) or spinal cord injury (SCI) may have profound, negative effects on sexual life [1]. Both conditions can induce many manifestations that interfere with sexual activity and sexual satisfaction. Impaired sexual functioning may lead further to isolation, embarrassment, social anxiety, loss of confidence or motivation, poor self-esteem, and depression, finally shrinking overall well-being and quality of life.

Vigorous concerted efforts of many different healthcare professionals are needed to ameliorate prospects and well-being of neurogenic patients. This goal requires thorough diagnosis and treatment of sexual dysfunction (SD) in this distinctive patient group. The European Association of Urology currently recommends the use of validated instruments to assess SD for patients with chronic neurogenic disorders because nonspecific instruments are not sufficiently sensitive [2]. Clinicians may underestimate patient symptoms in up to 40% of cases [3]; thus, patient-reported outcome measures, typically questionnaires designed exclusively for neurogenic patients, should be used to avoid interviewer bias. Further, with specific instruments, healthcare providers are more likely to initiate discussion and identify sexual concerns that otherwise may be hard to accomplish because of patient reluctance to talk about sexuality [4]. MS and SCI are complex, disabling conditions that must be managed by teams of healthcare professionals. With respect to sexual problems of MS and SCI patients, those professionals badly need dependable, sound, and specific evaluation tools.

The Multiple Sclerosis Intimacy and Sexuality Questionnaire (MSISQ-15) is a well-known, acknowledged, sensitive, and specific questionnaire designed to accurately evaluate symptoms of SD in MS individuals [5]. The MSISQ-15 is uncomplicated and reliable in evaluating the severity of SD symptoms; thus, it is used also for patients after SCI [6]. The MSISQ-15 evaluates three dimensions categorized as primary, secondary, and tertiary SD; therefore, it is an optimal and comprehensive measurement instrument to completely assess SD of the neurogenic population. The first version of MSISQ-15 was created and validated in English [5] and later successfully adapted cross-culturally to Persian [7], Portuguese [8], Dutch [6], and Italian [9]. The problems of impaired sexual satisfaction and sexual activity in persons with MS or SCI is increasingly being studied [10]. However, there is no adapted, validated Polish language edition of MSISQ-15 to evaluate SD in patients with neurological disease. Therefore, our goal was to translate and authenticate a Polish language version of the MSISQ-15 to support clinicians who treat neurogenic individuals and who execute SD research.

## Methods

The research ethics committee of our institution approved the study. The MSISQ-15 copyright owner provided written permission to develop and distribute a Polish version of the questionnaire. All patients were apprised about the study purpose and procedures, and all provided written informed consent to participate.

### Study design

This was a prospective (cohort) validation study. We recruited the study population from the Department of Urology of the Jagiellonian University Medical College, Krakow, Poland. We invited all adult MS and SCI individuals who were registered in a patient database. We excluded patients who had no sexual activity during the preceding 6 months, and patients with cognitive impairment, pre-existing major chronic illnesses, dementia, mental retardation, active malignancies, or Polish language difficulties. After translation (see later), MS and SCI individuals assessed the MSISQ-15 instrument during direct discussions. Then, other MS and SCI persons were requested to complete the Polish version of the MSISQ-15, and either the International Index of Erection Function (IIEF-15) or the Pelvic Organ Prolapse/Urinary Incontinence Sexual Questionnaire (PISQ-31), first as outpatients (test) then at home 2 weeks later (re-test). IIEF-15 and PISQ-31 were used as reference questionnaires. Demographic and clinical attributes were obtained from patient medical records and subjected to assessments with pre-specified outcomes that included validity (content and construct/criterion validity) and reliability (internal consistency and reproducibility). Standardized guidelines and international recommendations for translation, adaptation, and validation of questionnaires were followed [11, 12].

### Instruments

The MSISQ-15 is a self-administered instrument that investigates the effects of neurogenic symptoms on sexual activity and sexual satisfaction during a preceding 6-month period [5]. The questionnaire is a validated short form of the MSISQ-19 [13]. The MSISQ-15 consists of 15 items that cover three domains (five questions/domain): primary SD (symptoms resulting from a neurogenic disorder that directly affect sexual function (weakened genital sensation, erectile dysfunction, orgasm dysfunction, decreased vaginal lubrication, and loss of libido); secondary SD (symptoms that result from a neurogenic disorder that indirectly affect sexual function, such as fatigue, muscle tightness, weakness, spasticity, bladder and bowel dysfunction, lack of coordination, poor mobility, adverse effects from MS medications, cognitive difficulties, and numbness, pain, burning, or discomfort in non-genital body areas); and tertiary SD (psychological, emotional, and social aspects of a neurogenic disease that affect sexual function, including feeling less virile/feminine or unattractive, concern about partner satisfaction, and general insecurity about one’s sexuality). Items are addressed with a Likert-like scale from 1 (never) to 5 (always). The questionnaire has a possible score of 15 to 75 (sum of the 15 items), wherein the higher the score, the greater effect of SD symptoms on patient life. The instrument does not differentiate between specific and nonspecific SD; the scale is an overall measure of SD.

The IIEF-15 is a brief, reliable, and multidimensional scale for evaluation of male sexual function [14]. We chose the IIEF-15 as the benchmark (validation) instrument for men because it is used widely by Polish urologists, and it is validated in Polish, but not specifically for neurogenic individuals [14].

The PISQ-31 is a reliable, well-known, and comprehensive questionnaire to assess sexual functioning in women with pelvic organ prolapse and/or urinary incontinence [15]. Because the PISQ-31 is used widely in clinics in Poland, we used it as the reference (validation) instrument for women. The PISQ-31 is validated in Polish, but not specifically for neurogenic individuals [15].

### Translation

Two professional and avowed translators whose native language was Polish separately translated the questionnaire from English into Polish (forward-translation). The investigators compared the two translations, made slight corrections without changing the content, and settled on a provisional version. A similarly credentialed professional translator, blinded to the original English version of the questionnaire, translated the provisional version into English (back-translation). Then, a concurrence meeting produced the final Polish version of the MSISQ-15. The final version was evaluated based on semantic, idiomatic, experiential, and conceptual equivalence. Three staff urologists, one psychiatrist, one gynecologist, and one neurologist checked and agreed on the final Polish version of the questionnaire.

### Validation − validity

#### Content validity (cross-cultural adaptation process)

With direct discussions, 60 patients (30 individuals with MS and 30 with SCI) assessed the content validity (i.e., how closely a measure was relevant to, and representative of, the construct it was designed to measure) [11]. First, patients completed the Polish version of the MSISQ-15. Then, wording and content were discussed one-on-one with each individual. Researchers and patients subjectively evaluated the match between the questionnaire items and symptoms.

#### Construct/criterion validity

Construct/criterion validity (i.e., congruity of the questionnaire score and a gold standard or to another questionnaire that shared similar theoretical grounds for a relationship) was evaluated by analyzing the correlation between the Polish version of the MSISQ-15 and the reference questionnaires [11]. Notably, the score correlations of the MSISQ-15 to the IIEF-15 for men and the MSISQ-15 to PISQ-31 for women were determined by Pearson correlation coefficients (from − 1 = perfect negative correlation, via 0 = no correlation, to + 1 = perfect positive correlation).

#### Ceiling effects

Ceiling/floor effects, analyzed to determine sufficient variance in observations, were considered to occur if > 15% of participants had the highest or lowest possible score [11]. The ceiling/floor effects were calculated for the total and domain scores.

### Validation − reliability

#### Internal consistency

Internal consistency referred to intercorrelation of the questions; a parameter that verifies correlations between items within a subscale, domain, or questionnaire, i.e., whether the items gauged the identical fundamental construct. The Cronbach’s alpha was computed for the total score and for the three separate domains of the MSISQ-15 [16]. A Cronbach’s alpha higher than 0.7 indicated good internal consistency [11].

#### Reproducibility

Reproducibility referred to test-re-test reliability that analyzed whether the investigated instrument consistently replicated the result more than once in the same situation and population, i.e., produced repeated measurements. We ensured that there was not any type of treatment intervention with the patients between the two measurements. The reliability was computed from the intraclass correlation coefficient that confronted the initial MSISQ-15 score with the repeat MSISQ-15 score 2 weeks hence (ICC_2,1_) [16]. A coefficient of 0.7 or greater revealed good reproducibility [11].

#### Further statistics

Descriptive results for continuous data were measured as means ± standard deviations. For discrete data, we used counts and percent. Statistical significance was regarded as *p* < 0.05. Data analysis was conducted with SPSS Statistics software, version 24.0 (IBM Corporation, Armonk, NY, USA).

Following standard recommendations for instrument validation and other validation studies, initially, we sought sample sizes of 50 MS and 50 SCI persons [6, 11]. However, we elected to increase the sample size after a consult with two independent experts on patient-reported outcome measures and two Polish language specialists. This enlargement in the sample size was based on specific Polish language pronunciation, declensions, and free word order. The adopted methodology increased accuracy and yielded a smaller margin of error with improved identification of the outliers [17]. For the same reasons, during cross-cultural adaptation (i.e., content validation), we directly interviewed a minimum of 30 MS and 30 SCI individuals, although 10 persons from each group would have afforded adequate content validity.

Because the European Association of Urology recommends individual validations for specific neurogenic patient groups, we validated the MSISQ-15 independently with individual data presentations for the MS and SCI patients [2]. Although there are some similarities between the SD symptoms of MS and SCI patients, the overall symptom presentations and effects on the quality of life may have varied (e.g., impaired sensibility in MS versus frequent total loss in SCI; gradual deterioration of the primary disease in MS versus acute beginning in SCI). Thus, patients with MS and SCI portray two major populations of neurogenic individuals: non-traumatic and traumatic [18].

## Results

We invited 328 patients to adapt and validate the questionnaire but 29 persons were excluded following the exclusion criteria. Therefore, 299 patients participated in this study. From this group, thirty MS and thirty SCI individuals were selected randomly to assess content validity/perform cross-cultural adaptation with in-person interviews. Those 60 individuals were excluded from the later steps of validation to avoid interviewer bias. After evaluation of content validity, 299 patients completed the instruments at baseline (test phase). For unknown reasons, eight patients did not return the second set of questionnaires, two refused to complete the second MSISQ-15 questionnaire, one did not fully complete the second set of questionnaires, and one died from pneumonia. Two hundred twenty-seven (117 MS and 110 SCI) persons completed the second set of questionnaires (re-test phase) on average 19.8 (standard deviation 10.3) days after the first set of questionnaires; we included these 227 individuals in the final analysis. In the re-test phase, no patient disclosed a change in sexual functions nor did any patient receive new medication/treatment for their SD.

Table 1 shows demographic and clinical features of the study participants. Most persons with MS were women who had relapsing-remitting disease, and most SCI individuals were men with thoracic injury.

**Table 1:** Demographic and clinical characteristics of the included patients

	Multiple sclerosis (*n* = 117)	Spinal cord injury (*n* = 110)
***Age (years)***		
	44.2 ± 9.4	39.2 ± 9.9
***Sex***		
Men	35 (29.9%)	98 (89.1%)
Women	82 (70.1%)	12 (10.9%)
***Time since diagnosis of neurological disease (years)***		
	9.2 ± 8.3	12.9 ± 10.9
***Stadium/level of neurological disease***		
*Multiple sclerosis*		
Relapsing-remitting	87 (74.4%)	
Primary progressive	12 (10.2%)	
Secondary progressive	13 (11.1%)	
Missing stadium of	5 (4.3%)	
*Spinal cord injury*		
ASIA A		59 (53.6%)
ASIA B		20 (18.2%)
ASIA C		6 (5.5%)
ASIA D		22 (20.0%)
ASIA E		3 (2.7%)
Cervical level		25 (21.4%)
Thoracic level		81 (69.2%)
Lumbar level		11 (9.4%)
***Mobility***		
Fully ambulatory	42 (35.9%)	21 (19.1%)
Limited walking	63 (53.8%)	25 (22.7%)
Wheelchair bound	12 (10.3%)	64 (58.2%)
***MSISQ-15 scores***		
Total score	34.83 ± 11.99	43.58 ± 11.35
Primary domain	12.79 ± 4.04	17.23 ± 4.01
Secondary domain	11.23 ± 3.89	13.45 ± 5.11
Tertiary domain	10.81 ± 4.95	12.90 ± 5.23
***PISQ-31 scores***		
Total score	96.41 ± 13.86 (*n* = 90)	76.14 ± 11.76 (*n* = 18)
***IIEF-15 scores***		
Total score	35.95 ± 18.77 (*n* = 27)	42.17 ± 14.95 (*n* = 92)

Note: values are presented as mean ± standard deviation or numbers

### Validation − validity

#### Content validity (cross-cultural adaptation)

During direct discussions, MS and SCI individuals discerned a wording issue in the Polish translated instrument that necessitated a minor adjustment before finalizing the questionnaire (Supplement – Polish MSISQ-15). Included participants confirmed that all of the questions were essential to evaluate the spectrum of sexual problems related to their neurological disorders. All the individuals also generally found the Polish version of the MSISQ-15 understandable, clear, and easy to complete. They also underlined the need for various health-care professionals in Poland to employ an MSISQ-15-type questionnaire in routine clinical practice. Therefore, we concluded that the content validity was optimal.

#### Construct/criterion validity

We found a significant correlation between the total scores of MSISQ-15 (the higher score, the greater severity of symptoms, i.e., worse sexual function) and the total scores of IIEF-15 for men (the higher score, the lower severity of symptoms, i.e., better sexual function; *r* = − 0.487 for the test phase and *r* = − 0.456 for the re-test phase; see Table 2). Likewise, the total scores of MSISQ-15 correlated with the total scores of PISQ-31 for women (the higher score, the lower severity of symptoms, i.e., better sexual function; *r* = − 0.709 for the test phase and *r* = − 0.688 for the re-test phase). Thus, we concluded that the construct/criterion validity was adequate.

**Table 2:** Construct/criterion validity of the Polish version of MSISQ-15 – correlation of the MSISQ-15 scores with the PISQ-31 scores in women and IIEF-15 scores in men

	Test			Re-test		
	r	*p*-value	Number of patients	r	*p*-value	Number of patients
MSISQ-15 versus IIEF-15	−0.487	< 0.001	133	−0.456	< 0.001	133
MSISQ-15 versus PISQ-31	−0.709	< 0.001	94	−0.688	< 0.001	94

#### Ceiling/floor effects

We did not find ceiling and floor effects in our analysis. Only three MS patients reported the lowest possible total score, and no patient reported the highest possible score. Zero to 13 % of the MS individuals arrived at the lowest possible scores for the individual domains, and one to 5 % had the highest possible scores. In the SCI group, no patient achieved the lowest possible score, and two persons had the highest possible score. For the separate domain scores, zero to 4 % of the SCI individuals had the lowest possible, and one to 8 % had the highest possible.

### Validation − reliability

#### Internal consistency

The Cronbach’s alpha coefficients for the total MSISQ-15 from MS and SCI individuals were > 0.8 for test and re-test phase (Table 3). The internal consistencies for the specific domains of the instrument (primary, secondary, tertiary SD) were also satisfactory, with Cronbach’s alpha coefficients of > 0.7 in both study groups. Therefore, we concluded that the internal consistency of the Polish version of the MSISQ-15 was good.

**Table 3:** Internal consistency – Cronbach’s alpha (n_1_ = 117 patients with multiple sclerosis; n_2_ = 110 patients with spinal cord injury; n_3_ = 227 patients with either multiple sclerosis or spinal cord injury, i.e., all patients)

	Multiple sclerosis - Cronbach’s alpha coefficient		Spinal cord injury - Cronbach’s alpha coefficient		All patients - Cronbach’s alpha coefficient	
	Test	Re-test	Test	Re-test	Test	Re-test
MSISQ-15 total score	0.93	0.94	0.86	0.87	0.90	0.91
MSISQ-15 primary domain	0.81	0.82	0.73	0.71	0.77	0.76
MSISQ-15 secondary domain	0.77	0.83	0.82	0.81	0.79	0.82
MSISQ-15 tertiary domain	0.90	0.91	0.88	0.92	0.90	0.92

#### Reproducibility

The ICCs for correspondence of the total MSISQ-15 and the three domains (primary, secondary, tertiary SD) were all higher than 0.7 in MS and SCI patients, which indicated excellent reproducibility (Table 4). We did not find a difference between baseline and re-test. Therefore, we concluded that the reproducibility was optimal.

**Table 4:** Test-re-test reliability (reproducibility) with the interclass correlation coefficients (ICCs_2,1_). (n_1_ = 117 patients with multiple sclerosis; n_2_ = 110 patients with spinal cord injury; n_3_ = 227 patients with either multiple sclerosis or spinal cord injury, i.e., all patients)

	Multiple sclerosis - ICC_2,1_ (95% CI)	Spinal cord injury - ICC_2,1_ (95% CI)	All patients - ICC_2,1_ (95% CI)
MSISQ-15 total score	0.91 (0.80–0.95)	0.92 (0.83–0.96)	0.92 (0.86–0.96)
MSISQ-15 primary domain	0.93 (0.82–0.97)	0.90 (0.82–0.94)	0.93 (0.89–0.96)
MSISQ-15 secondary domain	0.78 (0.70–0.86)	0.85 (0.75–0.90)	0.82 (0.77–0.88)
MSISQ-15 tertiary domain	0.87 (0.79–0.93)	0.84 (0.73–0.92)	0.87 (0.80–0.92)

ICC_2,1_ measures the agreement between the patients’ initial score and the follow-up score (2 weeks)

*CI* confidence interval

## Discussion

This study provides the first comprehensive Polish questionnaire that evaluates sexual function in patients with neurogenic disorders. Whereas some extant instruments for this specific patient population are available in the Polish language, existing questionnaires evaluate primarily bladder problems [19–21]. The Polish version of the MSISQ-15 proved its reliability, validity, and consistency from individuals with MS and SCI. Thus, the MSISQ-15 can be used widely in research and clinical practice of different healthcare professionals (e.g., neurologists, urologists, psychiatrists, sexologists) in Poland to investigate the effects of symptoms of neurogenic disorders on an individual’s sexual activity and sexual satisfaction. This new instrument may also ease physician reluctance to discuss sexual problems with their MS and SCI patients who are often underevaluated during routine appointments.

Complex care for neurogenic individuals, particularly for MS and SCI persons, is multidimensional and multifactorial. Because neurological disorders may strongly affect sexual function, assessment of SD is an important aspect for these patients’ quality of life. Current recommendations advocate specific instruments for investigation of sexual function [2]. Specific instruments enable physicians to fully assess a disease-specific condition, support selection of appropriate treatments, ensure true comparisons with other studies, and expedite international multicenter trials. Because various patient-reported outcome measures are available to evaluate sexual function in neurologic patients, a recently published systematic review underlined the fact that strong evidence is available only for the MSISQ-15/- 19 instrument [22]. Therefore, we decided to create, adapt, and validate a Polish language MSISQ-15. Our investigation is also the first rigorous conversion of the MSISQ-15/-19 into a Slavic language, a language second only to Russian in the number of native speakers.

An asset of our study was the contributions of two consistent groups of individuals with two distinct neurogenic disorders, i.e., MS and SCI, with individual and independent adaptations and validations. Individuals with MS and SCI represent non-traumatic and traumatic neurologic dysfunctions. Although the pathophysiology of these two neurogenic conditions differ, there are some similarities between the effects of neurological symptoms on sexual functioning and between treatment methods. These similarities lead to a hypothesis that some instruments may perform well in different neurogenic populations. Thus, our study is as an example of this hypothesis because it showed clearly the measurement properties of the MSISQ-15 for two types of patients and because we used discrete analyses based on current guidelines and recommendations [2, 11]. In addition, our study included the greatest number of MS and SCI patients among international validation studies of MSISQ-15.

All patients confirmed content validity of the Polish version of the MSISQ-15 during in-person discussions. We substantiated optimal construct/criterion validity for both MS and SCI patients because the MSISQ-15 translated into Polish correlated significantly with the IIEF-15 for men and the PISQ-31 for women. Positive associations were also observed for the three specific domains of the MSISQ-15. Because of homogeneity of included patients and adequate sample size, we did not detect ceiling or floor effects [6]. Our validation analysis showed good internal consistency with Cronbach’s alpha coefficients greater than 0.8 for the MSISQ-15 total score and superior to 0.7 for its specific domains, thereby confirming high discriminative power. Finally, the test-re-test study with ICCs greater than 0.9 for the total score of MSISQ-15 and superior to 0.7 for its specific domains verified internal stability and reproducibility of the MSISQ-15 in both studied populations. Overall, our results are broadly comparable with the results of other validation studies of MSISQ-15 [6, 9]. The measurement properties we evaluated were also similar to properties presented in the development of the original instrument [5].

How widely applicable is the Polish version of the MSISQ-15? Koltuniuk et al. reported that more than half of MS patients were unsatisfied with their sex life, and most had significant sexual disorders [23]. Poland has a relatively high number of MS patients, approximately 110/100,000 inhabitants [24]. With the current Polish population of 38.5 million people, the prevalence of MS is approximately 42,000 persons. In SCI, up to 90% of affected individuals experience SD [25]. No study has described the prevalence of SCI in Poland, although Tederko et al. reported an incidence about two-fold lower than that of MS [25]. Therefore, the strict and accurate process of translation and validation that we conducted is justified by the numbers of potential persons who would benefit from the Polish edition of MSISQ-15. Together with the MSISQ-15, we also advise use of questionnaires designed specifically for neurogenic patients to evaluate their urological symptoms, signs, consequences, and urinary disorder-specific quality of life. Relevant questionnaires include Qualiveen, SF-Qualiveen, and the Neurogenic Bladder Symptom Score (NBSS) that were recently validated in the Polish language for both MS and SCI patients [19–21]. Therefore, after years without appropriate specific instruments available in Poland for this distinct group of patients, Polish physicians of different healthcare backgrounds can finally provide comprehensive urological and sexual assessment for MS and SCI individuals.

Our investigation should be also assessed in the context of its limitations. We did not evaluate the responsiveness of the Polish version of the MSISQ-15. This omission was due to short follow-up that we adopted to ensure adequate test-re-test analyses with no clinical interventions between the two measurements and to minimize possible symptom changes. In addition, we acknowledge that a correlation coefficient for interconnection between IIEF-15 and MSISQ-15 was lower than a correlation coefficient between PISQ-31 and MSISQ-15. Nonetheless, moderate correlations of some MSISQ-15 components have been described [5, 7, 9]. These different correlations may have arisen because the MSISQ-15 instrument more broadly and comprehensively assesses SD compared with reference instruments that were not designed specifically for neurogenic patients. For our analysis, an absolute gold standard instrument was unavailable. A reference inquiry form would be an instrument that is widely used, validated for neurogenic patients, available in Polish, and able to assess symptoms of SD. Because we did not identify an ideal reference questionnaire, we used two commonly used instruments (IIEF-15 and PISQ-31) for evaluation of SD as the gold standards already tested in the other validation studies of MSISQ-15 [6]. Further, MSISQ-15 is an overall measure of sexual dysfunction designed for people with MS; it includes MS-related and MS-unrelated sexual concerns [5]. We did not include non-neurogenic individuals as a control because, as indicated by Terwee et al., control individuals were not necessary for successful conversion [11]. Indeed, a control group was not used in development of the original questionnaire [5]. Our introduction of MSISQ-15 in the Polish language followed all required criteria with evaluation of standardized measurement properties for the translation, adaptation, and validation of an instrument as put forward by Terwee et al. [11]. The use of established recommendations is necessary to reduce the risk of introducing bias into a study that moves a questionnaire into a different language in a different setting and during a different time.

## Conclusions

In this study, following international and well-established recommendations, we successfully translated, adapted, and validated the Polish version of MSISQ-15. It is a valid, comprehensive, and reliable tool for evaluation of SD symptoms in MS and SCI patients. The instrument will promote improvement of the quality of rehabilitative intervention and assessment of the sexual lives of neurogenic patients. We recommend that clinicians and investigators use the Polish version of MSISQ-15 in clinical practice and research in Poland.

## Supplementary Information

**Additional file 1.** Polish MSISQ-15.

**Additional file 2.** English MSISQ-15.

## Data Availability

All data generated or analysed during this study are included in this published article [and its supplementary information files]. The datasets used and/or analysed during the current study available from the corresponding author on reasonable request.

## Abbreviations

ICC: Intraclass correlation coefficient
IIEF-15: International Index of Erection Function
MS: Multiple sclerosis
MSISQ-15: Multiple Sclerosis Intimacy and Sexuality Questionnaire
PISQ-31: Pelvic Organ Prolapse/Urinary Incontinence Sexual Questionnaire
SCI: Spinal cord injury
SD: Sexual dysfunction
