# Supplementary material for: The Multiple Sclerosis Intimacy and Sexuality Questionnaire (MSISQ-15): translation, adaptation and validation of the Polish version for patients with multiple sclerosis and spinal cord injury
Source: BMC Neurol. 2021 Mar 8;21:103. doi: 10.1186/s12883-021-02132-9 (PMC7938601; doi:10.1186/s12883-021-02132-9)
Supplement: Supplementary file 1 — Additional file 1. Polish MSISQ-15. [file 12883_2021_2132_MOESM1_ESM.pdf]

MSISQ-15: Kwestionariusz dotyczący wpływu stwardnienia rozsianego lub urazu rdzenia kręgowego na intymność i seksualność

INSTRUKCJE: Aby lepiej zrozumieć wpływ stwardnienia rozsianego lub urazu rdzenia kręgowego na intymność i seksualność, prosimy, aby w tym obejmującym 15 pytań kwestionariuszu ocenił Pan/Pani, w jaki sposób różne objawy stwardnienia rozsianego lub urazu rdzenia kręgowego zakłócały Pana/Pani aktywność seksualną i satysfakcję seksualną w ciągu ostatnich sześciu miesięcy. Odpowiedzi można udzielać umieszczając krzyżyk lub jakikolwiek inny znak w kwadratowym polu przy pytaniu, pod właściwą cyfrą. Nie ma dobrych ani złych odpowiedzi. Jeśli nie jest Pan/Pani pewien, jak odpowiedzieć na pytanie, prosimy wybrać najlepszą możliwą odpowiedź.

NA PRZESTRZENI OSTATNICH SZEŚCIU MIESIĘCY, MOJĄ AKTYWNOŚĆ SEKSUALNĄ LUB SATYSFAKCJĘ SEKSUALNĄ ZAKŁÓCIŁY NASTĘPUJĄCE OBJAWY:

	nigdy (1)	prawie nigdy (2)	czasami (3)	prawie zawsze (4)	zawsze (5)
1. napięcie mięśni lub skurcze ramion, nóg lub innych okolic ciała					
2. objawy z zakresu pęcherza moczowego lub układu moczowego					
3. objawy jelitowe					
4. drżenie lub trzęsienie rąk lub innych części ciała					
5. ból, pieczenie lub dyskomfort w ciele					
6. poczucie, że moje ciało jest mniej atrakcyjne					
7. poczucie bycia mniej męskim lub kobiecym z powodu stwardnienia rozsianego/urazu rdzenia kręgowego					
8. słabsze czucie lub drętwienie genitaliów					
9. strach przed odrzuceniem seksualnym z powodu stwardnienia rozsianego/urazu rdzenia kręgowego					
10. obawy o zaspokojenie seksualne mojego(-j) partnera(-ki)					
11. mniejsza pewność swojej seksualności z powodu stwardnienia rozsianego/urazu rdzenia kręgowego					
12. brak zainteresowania seksem lub brak pożądania					
13. mniej intensywne lub przyjemne orgazmy lub szczytowania					
14. zbyt długie osiąganie orgazmu lub szczytowania					
15. nieodpowiednie nawilżenie pochwy (kobiety)/ trudności w osiągnięciu lub utrzymaniu zadowalającej erekcji (mężczyźni)					

Pozycje podskali dotyczące pierwotnych zaburzeń seksualnych: 8, 12, 13, 14, 15;
pozycje podskali dotyczące wtórnych zaburzeń seksualnych: 1, 2, 3, 4, 5;
pozycje podskali dotyczące trzeciorzędowych zaburzeń seksualnych: 6, 7, 9, 10, 11.
