# Supplementary material for: The Multiple Sclerosis Intimacy and Sexuality Questionnaire (MSISQ-15): translation, adaptation and validation of the Polish version for patients with multiple sclerosis and spinal cord injury
Source: BMC Neurol. 2021 Mar 8;21:103. doi: 10.1186/s12883-021-02132-9 (PMC7938601; doi:10.1186/s12883-021-02132-9)
Supplement: Supplementary file 2 — Additional file 2. English MSISQ-15. [file 12883_2021_2132_MOESM2_ESM.pdf]

Multiple Sclerosis Intimacy and Sexuality Questionnaire-15 (MSISQ-15)

INSTRUCTIONS: In order to better understand the impact of multiple sclerosis on intimacy and sexuality, this 15-item questionnaire asks you to rate how various MS symptoms have interfered with your sexual activity or satisfaction over the last six months. Questions may be answered by placing a check or any other mark in the square located next to the question and below the appropriate number. There are no right or wrong answers. If you are unsure how to answer a question, please choose the best answer you can.

OVER THE LAST SIX MONTHS, THE FOLLOWING SYMPTOMS HAVE INTERFERED WITH MY SEXUAL ACTIVITY OR SATISFACTION:

	never (1)	almost never (2)	occasionally (3)	almost always (4)	always (5)
1. muscle tightness or spasms in my arms, legs, or body					
2. bladder or urinary symptoms					
3. bowel symptoms					
4. tremors or shaking in my hands or body					
5. pain, burning, or discomfort in my body					
6. feeling that my body is less attractive					
7. feeling less masculine or feminine due to MS					
8. less feeling or numbness in my genitals					
9. fear of being rejected sexually because of MS					
10. worries about sexually satisfying my partner					
11. feeling less confident about my sexuality due to MS					
12. lack of sexual interest or desire					
13. less intense or pleasurable orgasms or climaxes					
14. takes too long to orgasm or climax					
15. inadequate vaginal wetness or lubrication (women)/difficulty getting or keeping a satisfactory erection (men)					

Primary sexual dysfunction subscale items: 8, 12, 13, 14, 15; secondary sexual dysfunction subscale items: 1, 2, 3, 4, 5; tertiary sexual dysfunction subscale items: 6, 7, 9, 10, 11.
